# Are sleep and breakfast behaviours predictors of active or independent commuting to school in Spanish youth?

**DOI:** 10.1186/s12889-026-29160-9

**Published:** 2026-08-22

**Authors:** Ana Ramírez-Osuna, Francisco Javier Huertas-Delgado, Alberto Aibar-Solana, Palma Chillón, Pablo Campos-Garzón

**Affiliations:** 1 Department of Physical Education and Sports, Faculty of Sport Sciences, Sport and Health University Research Institute (iMUDS), University of Granada, Granada, Spain; 2 “La Inmaculada” Teacher Training Centre, University of Granada, Granada, 18013 Spain; 3 Departamento de Expresión Musical, Plástica y Corporal, Facultad de Ciencias Humanas y de la Educación, Universidad de Zaragoza, Huesca, España; 4 Department of Global Public Health, Karolinska Institutet, Stockholm, Sweden

**Keywords:** Active transport, Independent travel, Bedtime, Breakfast, Youth

## Abstract

**Background:**

Despite increasing interest in the association between active commuting to school (ACS) and health-related behaviors, the existing literature presents inconsistent findings regarding the relationships between ACS and preceding behaviors such as sleep duration and daily breakfast consumption. The current study aimed to analyze the associations between the mode of commuting to school and independent mobility to school with sleep duration and daily breakfast consumption among Spanish children and adolescents, and the secondary aim was to conduct this analysis separately by gender.

**Methods:**

It was a cross-sectional study conducted in 27 schools from 11 Spanish cities involving 226 children (6–11 years) and 1155 adolescents (12–17 years). Mode of commuting to school, independent mobility to school (yes/no), sleep duration (adequate/non-adequate), and daily breakfast consumption (yes/no) were self-reported. Multilevel mixed-effects logistic regressions examined these associations across age groups and gender.

**Results:**

The results showed that children with adequate sleep duration were more likely to actively commute to school (OR: 2.98, 95% CI: 1.06 to 8.40), with stronger associations in boys (OR: 6.26, 95% CI: 1.32 to 29.8). Regarding adolescents, those who consumed breakfast daily were more likely to commute independently to school (OR: 1.47, 95% CI: 1.05 to 2.07). No other significant associations were observed in children and adolescents (all, *p* > 0.05).

**Conclusions:**

Based on our findings, there may be a tendency for preceding behaviours, such as adequate sleep duration and daily breakfast consumption, to influence whether children and adolescents commute to school actively or passively, and whether they do so independently or with adult supervision. Programs promoting these behaviors should involve families, schools, and communities to support healthier lifestyles and enhance the well-being of children and adolescents.

**Supplementary Information:**

The online version contains supplementary material available at 10.1186/s12889-026-29160-9.

## Introduction

The global prevalence of overweight and obesity among young people increased by 5.6% in girls and 7.8% in boys between 1975 and 2016 [1]. Although overweight and obesity have multifactorial causes [2], The World Health Organization recommends that children and adolescents aged 5–17 engage in at least 60 min of moderate-to-vigorous physical activity (MVPA) per day [3]. Increasing physical activity (PA) levels is considered one of the most modifiable factors in addressing overweight and obesity [2]. Nevertheless, the current situation is alarming, with low adherence to these PA recommendations among both children and adolescents, particularly among girls [3]. In Spain, recent nationwide studies, such as the PASOS study and the Global Matrix 4.0 report, have shown that fewer than 50% of Spanish youth meet the daily PA recommendations [4, 5]. Considering these factors, it is necessary to implement interventions aimed at increasing PA levels in Spanish youth. Given that children and adolescents around the world typically commute to and from school at least 10 times per week, active commuting to school (ACS), primarily by walking or cycling, has been recognized as an effective strategy to increase daily PA and help meet the WHO recommendations for this age group [6]. Additionally, ACS has been directly associated with several health benefits, including improved cardiovascular health, lower body mass index, and enhanced cardiovascular fitness [7–9].

However, despite the well-documented benefits of ACS, global trends reveal substantial variation in its prevalence among youth [4, 5, 7], suggesting room for improvement. According to the Global Matrix 4.0 report [4], ACS rates range from 20% to 86%. Overall, ACS rates in Spain have maintained consistent patterns around 50%-60% [10], despite the interventions carried out in the last years to increase the rates of ACS in children and adolescents’ population [11, 12]. In addition, an important feature of ACS is that it can be performed independently [13]. Independent mobility to school, defined as commuting without adult supervision, has been associated with various health benefits in young people, including increased social interaction with peers [14], better spatial and traffic safety skills for ACS [15], or maturity regarding decision-making levels [16]. Nevertheless, the prevalence of independent mobility to school remains below 35% across Europe [17]. In Spain, recent studies have reported that the prevalence of independent mobility to school is approximately 35–40% among children and around 50% among adolescents, showing a clear increase with age [18, 19]. Therefore, the low rates of ACS and independent mobility to school raise concerns from both a developmental and public health perspective, as they reflect changing habits directly related to PA levels in this population. Previous studies have shown that ACS is influenced by several individual, family, and environmental factors, such as socioeconomic status, distance to school, parental commuting mode, and safety perceptions [10, 15, 20]. However, while most research has focused on these traditional correlates, little is known about pre-commute behaviors such as sleep and breakfast habits, for which existing evidence remains inconsistent [21, 22].

Since ACS is just one behavior within a broader healthy lifestyle, recent studies have examined the relationship between ACS and factors such as sleep duration and daily breakfast consumption [23–27]. Despite increasing interest in the association between ACS and health-related behaviors, the existing literature presents inconsistent findings regarding the relationships between ACS and preceding behaviors such as sleep duration and daily breakfast consumption. Studies conducted in Spanish adolescents have reported that adequate sleep is positively associated with ACS [21]; however, these studies did not include younger children and failed to stratify their results by gender. Notably, the study by Villa-González et al. [22] in Ecuadorian youth stratified results by gender and found that only adolescent girls with adequate sleep were more likely to engage in ACS. Regarding breakfast consumption, the evidence remains inconclusive: some studies indicate that adolescents who consume breakfast daily are less likely to commute actively [28], while others report no significant associations [21, 22]. Furthermore, no previous study has examined whether these preceding behaviors are associated with independent mobility to school, despite its strong predictive value for ACS. These limitations underscore the need for further research that includes both children and adolescents, adopts a gender-sensitive approach, and treats independent mobility as a central variable rather than a peripheral one. Moreover, contextual factors such as socioeconomic status, parental support, and distance to school are known to influence commuting behaviours and should be considered when interpreting these associations [10, 15]. Therefore, the main aim of the current study was to analyze the associations between the mode of commuting to school and independent mobility to school with sleep duration and daily breakfast consumption among Spanish children and adolescents, and the secondary aim was to conduct this analysis separately by gender.

## Methods

### Study design

This cross-sectional study was conducted in 27 schools (8 primary and 19 secondary) across 11 Spanish cities between March 2015 and June 2021. All schools were initially contacted by phone, followed by an in-person meeting between the research team and headteachers to explain the study’s objectives. Upon approval from each school, families provided signed informed consent for their children’s participation. Subsequently, children and adolescents who agreed to participate confirmed their consent with their Physical Education teacher. A specific date was then scheduled for data collection. On the appointed day, members of the research team visited each school and administered a paper-based questionnaire, which was completed within one hour. The study was approved by the Review Committee for Research Involving Human Subjects at the University of Granada (Reference: 162/CEIH/2016).

### Participants

**Fig. 1:**
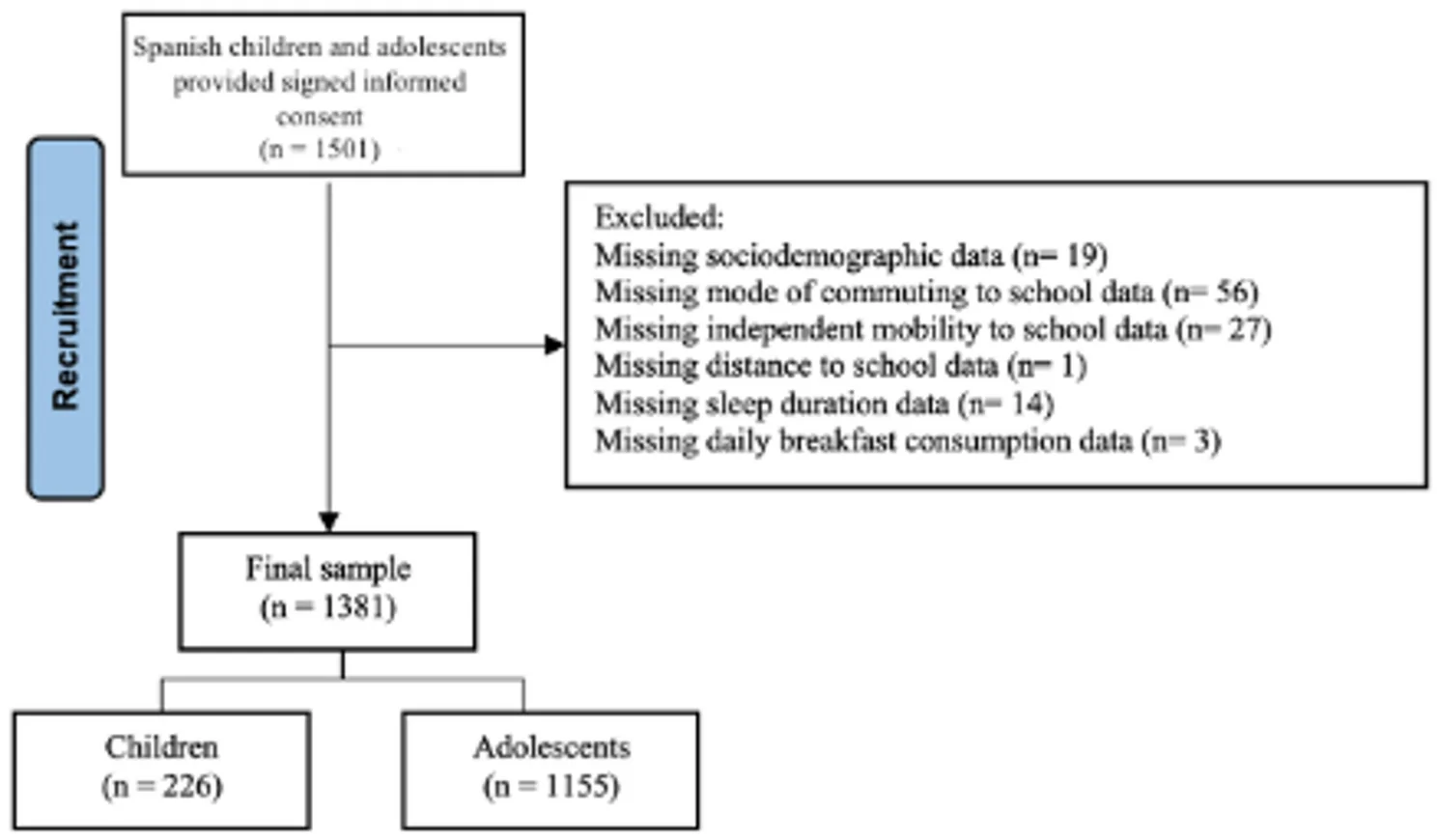
Flow chart of the participants

A total of 1,501 Spanish children and adolescents provided signed informed consent. After applying the inclusion criteria, (1) complete data on sociodemographic variables and distance to school; (2) complete data on the mode of commuting to school; (3) complete data on independent mobility to school; and (4) complete and valid data on sleep duration (i.e., time to go to bed and time to wake up) as well as daily breakfast consumption, a final sample of 1,381 participants was included in the statistical analysis. The proportion of missing data was minimal and therefore multiple imputation was not deemed necessary. Only participants with complete data for the main study variables were included in the analyses to ensure internal consistency and comparability across models. Of these, 226 were children (51.3% female; mean age: 11.0 ± 0.7 years) and 1,155 were adolescents (52.9% female; mean age: 14.0 ± 1.3 years) (Fig. 1).

### Measures

#### Sociodemographic data

Participants self-reported their gender and age. They were categorized as “children” if they were between 6 and 11 years old, and as “adolescents” if they were between 12 and 17 years old.

### Usual mode of commuting to school

Using a reliable and validated questionnaire [29], children and adolescents self-reported their usual mode of commuting by responding to the question (see in supplementary material): “How do you usually commute to school?” The questions on commuting mode and distance were based on the validated “New Version of the Mode and Frequency of Commuting to and from School” questionnaire [30] which demonstrated substantial to almost perfect reliability in Spanish youth. Response options included: “I didn’t go to school,” “walking,” “cycling,” “car,” “motorbike,” “school bus,” “public bus,” “train/metro,” and “other.” Participants who reported walking or cycling were categorized as engaging in active commuting to school, whereas those who reported commuting by car, motorbike, school bus, public bus, or train/metro were categorized as engaging in passive commuting to school. Modes reported as “other” were classified as active if they involved physically active transport (e.g., kick scooter); otherwise, they were classified as passive.

### Independent mobility to school

Children and adolescents were asked whether they commuted to school independently, using the same question for both age groups (see in supplementary material): “Who do you go to school with?” Response options included: “with my father,” “with my mother,” “with my friends,” “with my grandparents,” “alone,” “with my neighbors,” “with my brother or sister,” and “others.” In some versions, the options were further specified as: “with my grandfather,” “with my grandmother,” “with other children/adolescents,” or “with other adults.” Participants were classified as having independent mobility to school if they reported commuting “alone,” “with my friends,” “with my brother or sister,” or “with other children/adolescents.” Those who selected any adult accompaniment were categorized as having dependent mobility to school. If participants chose “other” and specified commuting with a minor, they were classified as independent; if they specified commuting with an adult, they were classified as dependent. In cases where multiple options were selected, including both a minor and an adult, participants were classified as having dependent mobility. Independent mobility to school was defined, in line with previous research, as commuting without adult supervision [13, 15, 18].

### Sleep duration

Children and adolescents self-reported, in hour format, the exact time they went to bed and the time they woke up on school days using the following questions (see in supplementary material): “What time do you go to sleep every day?” and “What time do you wake up every day?” The research team then calculated the total number of hours each participant spent sleeping. According to the Sleep Foundation, [31] children were categorized as having “adequate sleep duration” if they slept between 9 and 11 h, while adolescents were categorized as having “Adequate sleep duration” if they slept between 8 and 10 h. If they did not meet these recommendations, both children and adolescents were categorized as having “non-adequate sleep duration”.

### Daily breakfast consumption

Children and adolescents self-reported the number of days they typically had breakfast before commuting to school by answering the following question (see in supplementary material): “From Monday to Friday during the weeks you go to school, how many days do you usually have breakfast?” Response options included: “I never have breakfast on school days,” “1 day,” “2 days,” “3 days,” “4 days,” and “5 days.” Participants were classified as having daily breakfast consumption if they reported eating breakfast on all five days, and as skipping breakfast if they selected any of the other options.

### Distance to school

The distance to school was obtained using a self-reported questionnaire previously shown to have good test-retest reliability in Spanish children (6–12 years) and adolescents (12–17 years) [32]. Participants answered the following question (see Supplementary Material): “How far do you live from school?” Response options differed slightly between questionnaire versions. One set of options was: “<0.5 km”, “0.5 km to < 1 km”, “1 km to < 2 km”, “2 km to < 3 km”, “3 km to < 5 km”, and “>5 km”. The other set of options was: “<0.5 km”, “0.5 km to < 1.5 km”, “1.5 km to < 3 km”, “3 km to < 6 km”, and “>6 km”. Given these differences, distance was recategorized as “<0.5 km”, “0.5 km to < 3.0 km”, and “>3 km”. The questionnaire demonstrated substantial to almost perfect test-retest agreement for the distance item (κ = 0.75 in children and κ = 0.90 in adolescents), indicating good stability of responses over time, although these values do not necessarily reflect the accuracy of the reported home-to-school distance.

### Statistical analysis

Descriptive statistics were reported as means ± standard deviation (SD) for continuous variables, and as frequencies and percentages for categorical variables. Normality was assessed using the Kolmogorov–Smirnov test and Q–Q plots. Chi-square tests and Mann–Whitney U tests were conducted to examine differences by age, mode of commuting to school, independent mobility to school, distance from home to school, sleep duration, and daily breakfast consumption across age groups and gender. Given the hierarchical structure of the data (participants nested within schools, and schools within cities), multilevel analysis was deemed appropriate. For all models, a nested random effects structure was specified, schools (ACS ICC: 0.190; independent mobility ICC: 0.258) nested within cities (ACS ICC: 0.202; independent mobility ICC: 0.210), as the likelihood ratio test indicated that this structure performed better than a random intercept model with only the school level (ACS ICC: 0.208; independent mobility ICC: 0.269). Accordingly, three mixed-effects logistic regression models were conducted: (1) one for the total sample, (2) one for children, and (3) one for adolescents. These models, implemented using the lme4 R package, analyzed the associations between the mode of commuting to school and both sleep duration and daily breakfast consumption, with stratified analyses by age group and gender. The same modeling procedure was applied to examine the associations between independent mobility to school and the two behaviors. In all models, mode of commuting and independent mobility to school were treated as outcome variables, while sleep duration and daily breakfast consumption were included as fixed effects in separate models for each behavior. All models were adjusted for age, gender, and self-reported distance from home to school. Moreover, the model discrimination was evaluated by plotting Receiver Operating Characteristic (ROC) curves and calculating the Area Under the Curve (AUC) using the pROC package. These analyses have been included in the supplementary material. We also explored including mode of commuting as an additional covariate in the models where independent mobility to school was the outcome. However, because mode of commuting and independent mobility represent closely related behavioural constructs, including both variables in the same model was considered likely to introduce conceptual overlap and potential over-adjustment. Therefore, mode of commuting was not included in the final models. The level of statistical significance was set at *p* *<* 0.05.

## Results

**Fig. 2:**
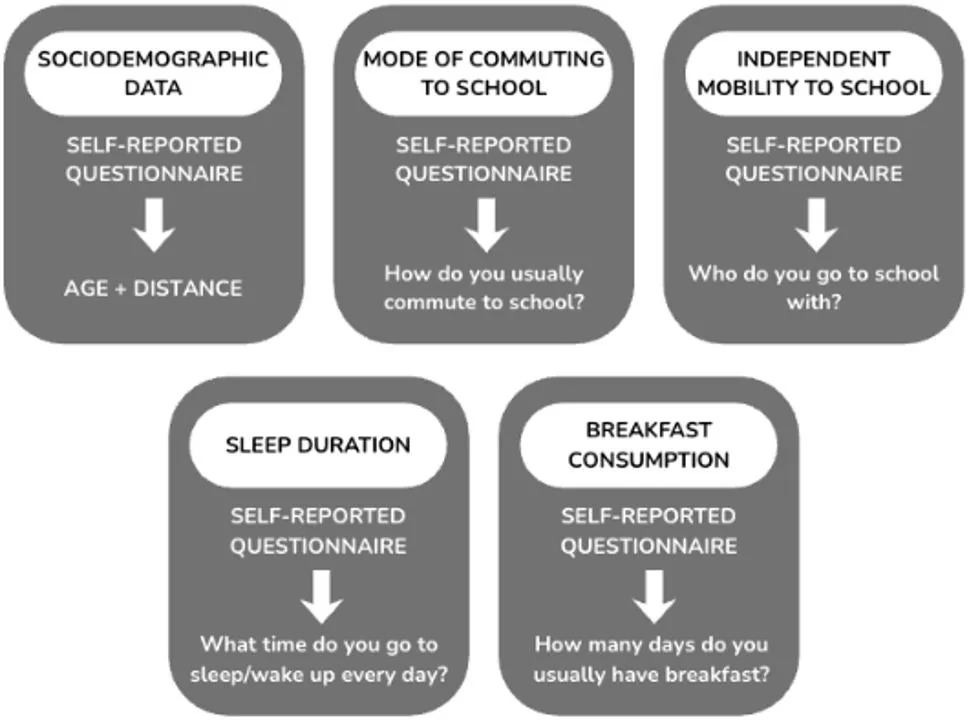
Measures among children and adolescents

**Fig. 3:**
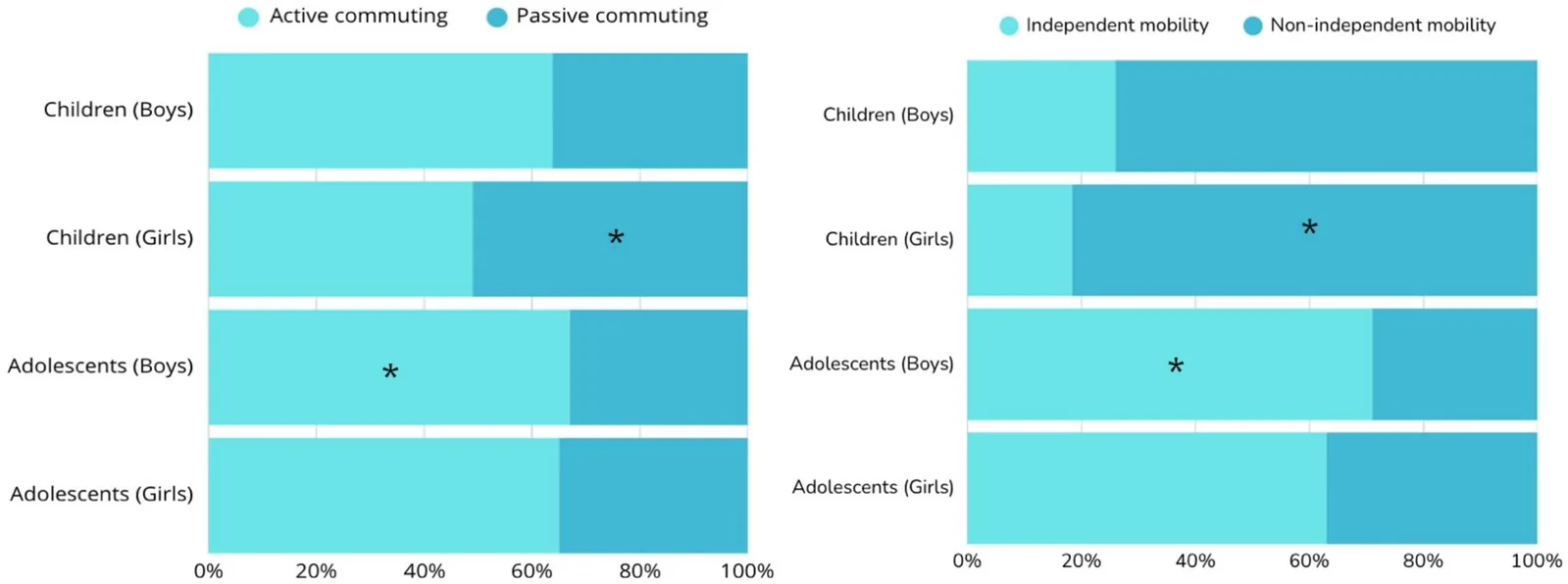
Mode of commuting and independent mobility among children and adolescents by gender. Note: Based on the total sample (*n* = 1,381). Asterisks indicate statistically significant differences between groups

**Fig. 4:**
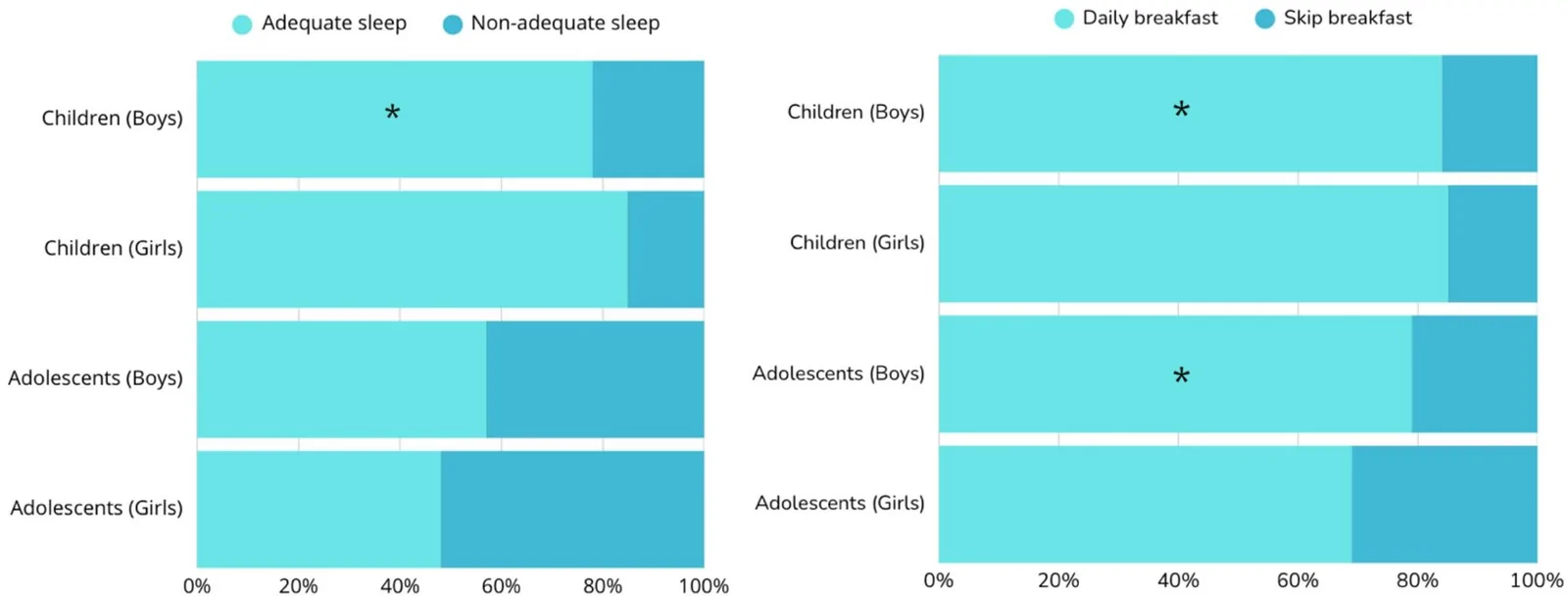
Sleep duration and breakfast consumption among children and adolescents by gender. Note: Based on the total sample (*n* = 1,381). Asterisks indicate statistically significant differences between groups

Descriptive data of the participants are summarized below and illustrated in Figs. 2, 3 and 4. Among the child participants, more than 50% actively commuted to school, while fewer than 25% commuted independently. Boys were more likely to use active modes of commuting compared to girls (63.6% vs. 49.1%; *p* = 0.03) and showed a higher prevalence of independent mobility to school than girls (26.4% vs. 19.8%; *p* = 0.031). Regarding the distance from home to school, most children lived within three kilometers of their school (81.9%). Additionally, children tended to have adequate sleep duration and consume breakfast daily (81.4% and 84.5%, respectively). No differences were found between genders in distance to school, sleep duration, or daily breakfast consumption among children (all, *p* > 0.05). In the adolescent population, more than 65% actively commuted to school, and 66.7% commuted independently (boys: 70.8% vs. girls: 63.0%; *p* < 0.01). Both boys and girls lived within three kilometers of their school (82.9% and 78.9%, respectively; *p* > 0.05). Most adolescents reported daily breakfast consumption (73.6%), and about half had adequate sleep duration (52.5%). Significant gender differences were found in sleep duration (boys: 57.4% vs. girls: 48.1%; *p* < 0.01) and daily breakfast consumption (boys: 79.2% vs. girls: 68.6%; *p* < 0.01). Interaction analyses were also conducted to examine whether associations between sleep duration or breakfast consumption and commuting behaviors differed by gender. No statistically significant interactions were observed in either children or adolescents (*p* > 0.05).

Associations between the mode of commuting to school and both sleep duration and daily breakfast consumption for children and adolescents are shown in Table 1. Children with adequate sleep duration were more likely to actively commute to school (OR: 2.98; *p* = 0.02). In the gender-specific analysis, boys were significantly more likely to actively commute if they had adequate sleep (OR: 6.26; *p* < 0.001), whereas no significant associations were found for girls (*p* > 0.05). No associations were found between mode of commuting and daily breakfast consumption in children, nor in the gender-specific analyses (all, *p* > 0.05). Among adolescents, no associations were found between mode of commuting and either adequate sleep duration or daily breakfast consumption, including in gender-specific analyses (all, *p* > 0.05).

**Table 1:** Association between mode of commuting to school* and sleep duration and daily breakfast consumption in children and adolescents

	Children			Adolescents		
	All (*n* = 226)	Boys (*n* = 110)	Girls (*n* = 116)	All (*n* = 1155)	Boys (*n* = 544)	Girls (*n* = 611)
Sleep duration (Ref: Non-adequate sleep)						
Adequate sleep	**2.98** **(1.06**,** 8.40)**	**6.26** **(1.32**,** 29.80)**	1.11 (0.23, 5.33)	0.81 (0.57, 1.15)	0.92 (0.55, 1.54)	0.75 (0.46, 1.22)
Daily breakfast consumption (Ref: Skip breakfast)						
Daily breakfast	2.54 (0.87, 7.42)	3.52 (0.75, 16.5)	1.97 (0.40, 9.72)	1.15 (0.78, 1.70)	0.91 (0.50, 1.68)	1.35 (0.81, 2.24)

Results presented as OR (95% confidence interval). All models were adjusted by distance from home to school and age. “All” in children and adolescents was also adjusted by gender

*Ref: Passive commuting to school

Table 2 displays the associations between independent mobility to school and both sleep duration and daily breakfast consumption in children and adolescents. No associations were found between independent mobility to school and either sleep duration or breakfast consumption among children, nor in the gender-specific analyses (all, *p* > 0.05). In adolescents, a positive association was observed between daily breakfast consumption and independent mobility to school (OR: 1.47; *p* = 0.03). No additional associations were found between independent mobility and sleep duration in adolescents, nor in the gender-specific analyses for either sleep duration or daily breakfast consumption (all, *p* > 0.05).

**Table 2:** Association between independent mobility to school* and sleep duration and daily breakfast consumption in children and adolescents

	Children			Adolescents		
	All (*n* = 226)	Boys (*n* = 110)	Girls (*n* = 116)	All (*n* = 1155)	Boys (*n* = 544)	Girls (*n* = 611)
Sleep duration (Ref: Non-adequate sleep)						
Adequate sleep	1.18 (0.51, 2.76)	1.26 (0.40, 3.98)	1.17 (0.32, 4.26)	1.33 (0.98, 1.81)	1.31 (0.84, 2.04)	1.36 (0.88, 2.10)
Daily breakfast consumption (Ref: Skip breakfast)						
Daily breakfast	1.31 (0.54, 3.16)	0.90 (0.26, 3.08)	1.74 (0.52, 5.83)	**1.47** **(1.05**,** 2.07)**	1.41 (0.83, 2.40)	1.56 (0.99, 2.45)

Results presented as OR (95% confidence interval). All models were adjusted by distance from home to school and age. All category was also adjusted by gender

*Ref: Non-Independent mobility to school

A visual summary of previous findings regarding the associations between sleep duration, breakfast consumption, and commuting behaviors can be found in the supplementary material (Figure S25 and Figure S26).

## Discussion

The main aim of the current study was to analyze the associations between the mode of commuting to school and independent mobility to school with sleep duration and daily breakfast consumption among Spanish children and adolescents, with a secondary aim of conducting this analysis separately by gender. The main findings showed that children with adequate sleep duration were nearly three times more likely to actively commute to school, with even stronger associations observed among boys, who were more than six times as likely to do so compared to boys with inadequate sleep duration. Moreover, our findings indicated that adolescents who consumed breakfast daily were 47% more likely to commute independently to school. No further associations were found between mode of commuting or independent mobility and sleep duration or daily breakfast consumption in either children or adolescents. Therefore, these preceding behaviors, adequate sleep duration and daily breakfast consumption, may influence whether children and adolescents actively or independently commute to school. Researchers and practitioners aiming to promote ACS and independent mobility should consider integrating these aspects into future intervention programs, although further research is needed to confirm these associations.

The current study showed that children with adequate sleep were more likely to use active modes of commuting to school, particularly boys. While these findings cannot be directly compared to other Spanish studies, since those primarily focused on adolescent populations, they differ from the study conducted by Villa-González et al. [22]. In their study, no association was reported between ACS and sleep duration among children. One possible explanation for these findings could be related to the significant role of parents in deciding the mode of commuting to school, particularly for children, it is ultimately the parents who determine how their child commutes. If they perceive their child as rested and refreshed, they may be more likely to opt for an active mode of commuting [33]. This hypothesis could be supported through the Theory of Planned Behavior [34], children who are well rested may show stronger intentions to actively commute, reinforced by parental encouragement and the perception that being active is a valued behaviour [35]. Another relevant finding was that daily breakfast consumption was not associated with ACS in children. This result is supported by a previous study conducted in Ecuadorian children, which also found no positive association between ACS and breakfast consumption [22]. Children may spend more time having breakfast in the morning, which could limit the time available for ACS [21, 22, 36]. These findings suggest that family routines around breakfast may influence not only breakfast consumption but also children’s daily PA habits, assuming that the breakfast routine is prioritized over the use of active modes of commuting to school. This explanation aligns with the 24-hour movement behavior framework [37], which emphasizes that daily behaviors such as sleep, PA, sedentary time, and eating routines are interdependent and constrained within the finite 24h of a day. Further research is needed on this topic to confirm these results, including parents as well as focus groups to understand the actual influence of these behaviors that precede ACS.

On the other hand, no associations were found between ACS and either sleep duration or daily breakfast consumption in adolescents. Regarding sleep duration, these results differ from those reported in previous studies conducted among Spanish adolescents, where adequate sleep duration was associated with the use of active modes of commuting to school [21, 28, 38]. Nevertheless, similar to the current study, Villa-González et al. [22] did not find associations between ACS and sleep duration for boys, but girls were more likely to use active modes of commuting if they had adequate sleep. According to Self-Determination Theory [39], motivation can be influenced by both individual and interpersonal factors, conditions that can be shaped by routines like sleep duration or daily breakfast consumption in ACS. Thus, as children grow older, they acquire greater autonomy in their daily routines, including decisions about commuting, sleep duration and breakfast consumption. Another relevant finding in our study was that daily breakfast consumption was not associated with ACS. Regarding breakfast consumption, previous studies support our findings [21, 22]. However, the study carried out by Martinez-Gomez et al. [36] reported that Spanish adolescents who consumed breakfast daily were less likely to ACS. A possible explanation for our findings could be that adolescents classified as passive commuters may have more predictable morning schedules, allowing them more time for breakfast. Additionally, it is possible that, similar to children, adolescents may prefer to spend their time before school eating breakfast rather than engaging in ACS [21, 22, 36]. Further research is needed to support these findings by considering important factors that may moderate or mediate this association (e.g., social support, built environment characteristics).

Finally, no associations were found between independent mobility to school and either sleep duration or breakfast consumption in children. These findings may be explained by the fact that previous studies have shown that children who actively commute to school are more likely to do so independently, and this behavior may be influenced more by family-related factors than by adequate sleep or daily breakfast consumption [33]. Indeed, previous studies have shown that restrictions on children’s independent mobility to school are largely due to parental concerns about road safety and social dangers [15], as well as perceived traffic danger [40, 41], rather than children’s own habits. Also, previous research has shown that parents often prefer to drive their children to school, particularly during primary and early secondary education, due to safety concerns and time constraints [30]. Similar patterns have also been observed in Spain, where parental perceptions of safety strongly influence children’s commuting mode [33]. On the other hand, associations were found between independent mobility to school and daily breakfast consumption in adolescents. These associations may be explained by the fact that having a structured morning routine, including breakfast, can facilitate or encourage independent mobility [42]. Adolescents who have internalized the habit of eating breakfast daily may exhibit more disciplined time management, enabling them to plan their journey to school in a more independent and autonomous manner [33]. From the perspective of Comprehensive Action Determination Model [43], such routines can be understood as habits shaped by intentions, personal norms, and situational factors. For example, valuing independence (personal norm) and following stable morning practices may encourage adolescents to commute on their own. However, further research is needed, as this association may be influenced by other factors not controlled for in the present study, such as parental autonomy support, which has been shown to be a key determinant of adolescents’ independent mobility. [13, 44, 45], or new friendships which may cause adolescents to be more autonomous and not the fact of eating breakfast [13, 44, 45]. Beyond these individual-level explanations, ACS aligns with the socio-ecological model [46], which highlights that behaviors are shaped by multiple levels of influence. While this study focused on individual factors such as sleep and breakfast habits, these are inevitably affected by interpersonal, environmental, and policy-related factors that can either support or limit active and independent commuting.

### Practical implications

The results of this study highlight the need to promote healthy habits from a broader perspective in children and adolescents to support active and independent commuting to school. Families and schools can collaborate to teach the importance of good sleep habits through simple routines and awareness programs. The promotion of healthy behaviors, such as sufficient sleep and regular breakfast consumption, should involve coordinated efforts among families, schools, and communities, for example, through school-based health education initiatives that encourage healthier lifestyles. Moreover, the findings of this study suggest important directions for future research. It is essential to explore parental perspectives, the role of the physical and social environment, and factors such as distance to school, as these may significantly influence both active commuting and independent mobility. Cross-cultural studies are also needed to assess whether cultural or socioeconomic differences affect these behaviors. In addition, future research should further examine contextual and socioeconomic factors, such as vehicle ownership, household income, parental education, and perceived safety, which are known to play a fundamental role in shaping commuting patterns. These insights can help design effective programs that promote healthy habits and support active and independent commuting, ultimately improving the health and well-being of children and adolescents. Special attention should be given to promoting ACS among girls by addressing parental safety concerns and improving route safety. Previous research has shown that girls are more often driven to school and less likely to commute independently, mainly due to parental perceptions of safety [15]. Actions aimed at parents are key to changing these attitudes and increasing confidence in the safety of ACS. School and community programs that provide safe walking routes, visible adult supervision, and group commuting initiatives can also help. Therefore, gender-sensitive education that encourages autonomy and confidence in girls could help close the gap in ACS.

### Limitations and strengths

The main limitation of this study lies in its observational design, as the associations observed do not imply causality. In addition, the mode of commuting to school, independent mobility, sleep duration, daily breakfast consumption, and distance were all self-reported, which may have led to slight under- or overestimation. As with other self-reported behaviors, these measures could be influenced by recall bias or limited temporal awareness, particularly among younger participants. However, since the questions referred to usual or habitual behaviors rather than single-day events, the potential for recall errors may be reduced, improving reporting accuracy [47]. This approach minimizes day-to-day variability and supports the reliability of children’s responses even at younger ages. Nevertheless, some minor inaccuracies may have occurred when reporting bedtime and wake-up times, perceptions of distance to school, or even recalling whether breakfast was consumed. Such potential misreporting should be considered when interpreting the results. Future studies should employ objective devices to assess sleep duration and use geographic information systems to measure commuting distance more accurately and reduce potential bias. Although the questionnaire has demonstrated good test-retest reliability, its criterion validity against objectively measured home-to-school distance has not been established. Therefore, self-reported distance should be interpreted as a stable rather than an objectively accurate measure. Collecting parental information, such as education level or home address, could also provide more valid measures of socioeconomic status and home–school distance. Despite the large sample size, the sample is not representative, and important factors such as socioeconomic status and environmental characteristics were not included. This limitation is partly due to the use of different versions of the questionnaire, which did not contain SES-related items. However, the participating schools were mainly located in middle- to high-income areas, which reduces, although does not eliminate, the likelihood of large socioeconomic disparities within the sample. Another limitation relates to the absence of parental data, which could have provided complementary and more accurate information about children’s commuting behavior, home–school distance, and socioeconomic background. However, involving families in school-based studies presents important logistical challenges, including low parental participation rates and privacy concerns [48]. These challenges are even greater in multicity studies such as the present one. Future research, even with smaller samples, should aim to include parents to better capture family-level and contextual determinants of active and independent commuting to school.

Consequently, the commuting behaviors observed are expected to reflect family preferences and routines rather than necessity-driven choices, as described in the “necessity- versus choice-based physical activity models” framework [20]. Furthermore, the findings of this study may not be generalizable to other populations due to geographical and cultural differences between countries. Future studies should include representative samples and account for socioeconomic and environmental variables in order to expand and refine these findings. In addition, studies involving parents and focus groups could help assess the actual impact of preceding behaviors on active and independent commuting to school. Another limitation relates to the categorization of commuting mode. Due to the low representation of some categories (e.g., cycling, public transport), the sample lacked statistical power to apply multinomial or nested logit models. For this reason, commuting was analyzed using a dichotomous classification (active vs. passive). Additionally, although public transport involves short walking distances, all motorized modes were classified as passive commuting for consistency with previous research on commuting and other health-related behaviors such as sleep and breakfast routines [21, 22, 36]. However, future studies should consider mixed-mode commuting (i.e., combinations of active and motorized transport) as a distinct behavior, given its unique characteristics compared with purely active or passive commuting patterns. On the other hand, this study has several strengths that should also be highlighted. To the best of our knowledge, this is the first study to analyze the associations between independent mobility to school, sleep duration, and daily breakfast consumption among Spanish children and adolescents. In addition, the sample size was relatively large compared to previous studies that have examined the association between ACS and other health-related behaviors. The Spanish context has shown that distance to school is one of the main predictors of ACS [49], unlike many previous studies on ACS that do not adjust for this variable, our analysis included self-reported distance to school.

## Conclusion

Promoting adequate sleep duration and daily breakfast consumption may play a role in encouraging ACS and independent mobility to school among Spanish youth. Spanish children who had adequate sleep duration were more than twice as likely to use active modes of commuting to school. Notably, boys were six times more likely to engage in ACS if they had adequate sleep duration. Additionally, Spanish adolescents who consumed breakfast daily were more likely to commute to school independently. However, no further significant associations were found between modes of commuting or independent mobility to school and sleep duration or daily breakfast consumption in either children or adolescents, nor in gender-specific analyses. Future studies should examine these associations in diverse contexts, considering parental barriers and permissions and using focus groups to better understand underlying influences. Beyond the specific associations identified, this perspective underscores the need to address ACS and independent mobility from a comprehensive, multi-behavioral approach. Therefore, schools, families, and communities must work collaboratively to integrate health promotion into educational and social programs.

## Supplementary Information

Supplementary Material 1.

Supplementary Material 2.

## Data Availability

The data underlying this article cannot be shared publicly due to Spanish legislation as well as the privacy of individuals that participated in the study. However, by contacting the corresponding author, data code for the analyses can be provided upon reasonable request.
