# Supplementary material for: Are sleep and breakfast behaviours predictors of active or independent commuting to school in Spanish youth?
Source: BMC Public Health. 2026 Aug 22;26:2415. doi: 10.1186/s12889-026-29160-9 (PMC13499306; doi:10.1186/s12889-026-29160-9)
Supplement: Supplementary file 1 [file 12889_2026_29160_MOESM2_ESM.pdf]

DeepL

Subscribe to DeepL Pro to translate larger documents.
Visit www.DeepL.com/pro for more information.

STUDENT QUESTIONNAIRE

Proyecto PACO

Hello! We would like you to complet[...] thank you very much for your cooperation. Unless otherwise indicated, only one answer should be marked for each question.

It is important that **you answer all questions** using a blue or black pen, filling in the boxes for your chosen answer **completely**, as shown in the image. Thank you very much.

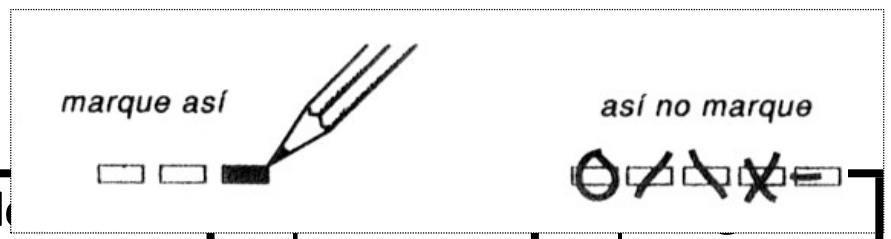

Today's date					
Day		Month		Year	
$$		$$		$$	
0U	0U	0U	0U	0U	0U
1U	1U	1U	1U	1U	1U
2U	2U		2U	2U	2U
3U	3U		3U	3U	3U
	4U		4U	4U	4U
	5U		5U	5U	5U
	6U		6U	6U	6U
	7U		7U	7U	7U
	8U		8U	8U	8U
	9U		9U	9U	9U

Start time (24-hour format)			
$$		: $$	
0U	0U	0	0U
1U	1U	1U	1U
2U	2U	2U	2U
	3U	3U	3U
	4U	4U	4U
	5U	5U	5U
	6U		6U
	7U		7U
	8U		8U
	9U		9U

Centre	
$$	
0U	0U
1U	1U
2U	2U
3U	3U
4U	4U
5U	5U
6U	6U
7U	7U
8U	8U
9U	9U

Cod			
☐	☐	☐	☐
0U	0U	0U	0U
1U	1U	1U	1U
2U	2U	2U	2U
3U	3U	3U	3U
4U	4U	4U	4U
5U	5U	5U	5U
6U	6U	6U	6U
7U	7U	7U	7U
8U	8U	8U	8U
9U	9U	9U	9U

$$	
0U	0U
1U	1U
2U	2U
3U	3U
4U	4U
5U	5U
6U	6U
7U	7U
8U	8U
9U	9U

$$	
	0U
1U	1U
2U	2U
3U	3U
4U	4U
5U	5U
6U	6U
7U	7U
8U	8U
9U	9U

Date of birth					
Day		Month		Year	
$$		$$		$$	
0U	0U	0U	0U	0U	0U
1U	1U	1U	1U	1U	1U
2U	2U		2U	2U	2U
3U	3U		3U	3U	3U
	4U		4U	4U	4U
	5U		5U	5U	5U
	6U		6U	6U	6U
	7U		7U	7U	7U
	8U		8U	8U	8U
	9U		9U	9U	9U

Level	
1UPrimary	
2USecondary	
3U Sixth Form	
Year	Group
U	U
1U	AU
2U	BU
3U	CU
4U	DU
5U	EU
6U	FU

You are
1UNiño
2UNiña

In which country were you born?		
	Spain	Other (please specify):
you?	U	U ______________________
**In which country was ... born?**		
	Spain	Other (please specify):
your mother?	U	U ______________________
Your father?	U	U ______________________

First and Last Names (CAPITAL LETTERS):

Telephone: **Email:**

Postal address (CAPITAL LETTERS):
(Street, number, flat, letter)

Town/City: **Postcode:**

Proyecto PACO

EDUCATIONAL CENTRE REFERS TO YOUR SCHOOL OR INSTITUTE

1. How far do you live from the educational centre?

Less than 0.5 km	From 0.5 to less than 1.5 km	From 1.5 to less than 3 km	From 3 to less than 6 km	6 kilometres or more
U	U	U	U	U

2. How long does it take you to get to school from your home?

Less than 5 minutes	From 5 to less 15 minutes	From 15 to less than 30 minutes	From 30 minutes to less than 60 minutes	60 minutes or more
U	U	U	U	U

3. How <u>do you usually get</u> to school? (<u>Select only one option</u>. If you combine several modes of transport, indicate the one that takes the most time)

Walking	By bicycle	Car	Motorcycle	Bus School	Public Public	Metro/ Train/ Tram	Other: (please specify)
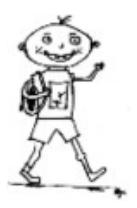	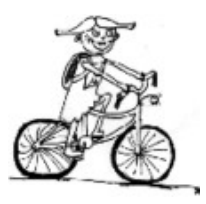	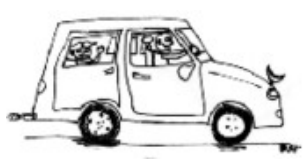	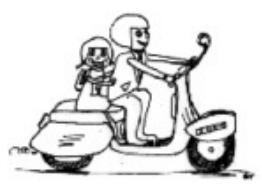	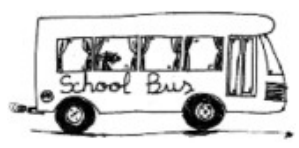	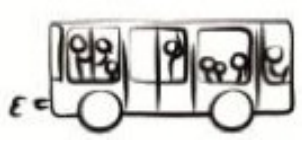	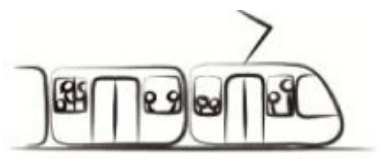	________
U	U	U	U	U	U	U	U

4. How <u>do you usually</u> get home? (<u>Select only one option</u>. If you combine several modes of transport, indicate the one you spend the most time on)

On foot	By bicycle	Car	Motorcycle	Bus School	Public Public	Metro/ Train/ Tram	Other: (please specify)
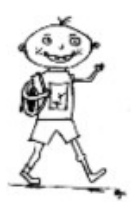	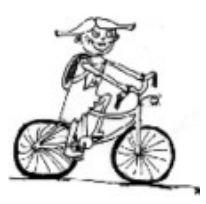	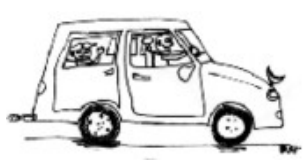	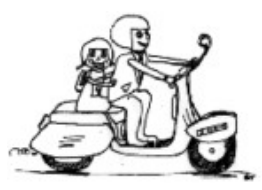	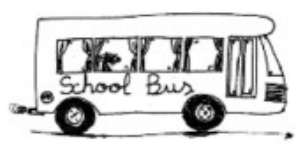	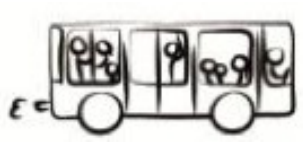	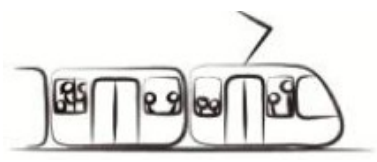	________
U	U	U	U	U	U	U	U

5. How many computers (desktops or laptops) do you have at home?

None	One	Two	More than two
U	U	U	U

6. Do you have any four-wheeled motor vehicles (car, van, lorry or motorhome) at home?

None	Yes, one	Yes, two or more
U	U	U

7. Do you have a bicycle in good working order?

Yes U No U

Think about the last 5 days you have had class (not including today) and answer questions 8 and 9.

8. **How did you travel to school each day? You can select more than one answer for each day if you used several means of transport to get to school.**

	Monday	Tuesday	Wednesday	Thursday	Friday
I didn't go to the city centre	U	U	U	U	U
Walking	U	U	U	U	U
Bicycle	U	U	U	U	U
Car	U	U	U	U	U
Motorcycle	U	U	U	U	U
School bus	U	U	U	U	U
Public bus	U	U	U	U	U
Metro/Train/Tram	U	U	U	U	U
Others: ______________ (please specify)	U	U	U	U	U

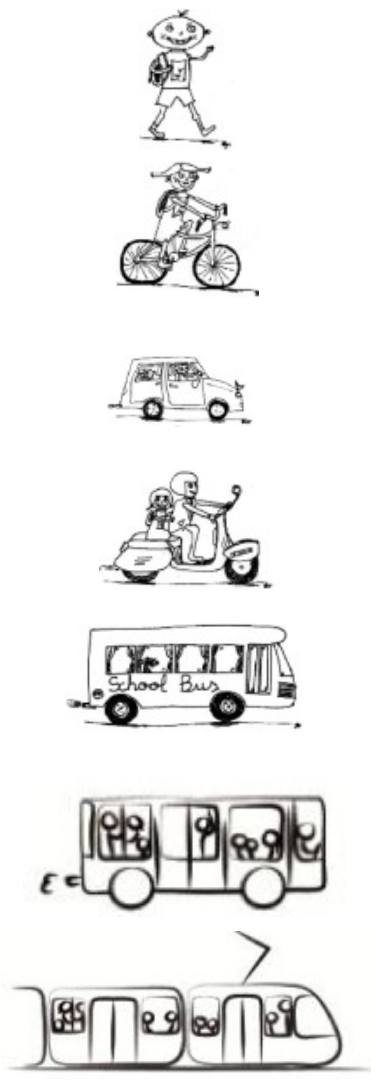

9. **How did you get home each day? You can select more than one answer for each day if you used several means of transport to get home from school.**

	Monday	Tuesday	Wednesday	Thursday	Friday
I didn't go downtown	U	U	U	U	U
Walking	U	U	U	U	U
Bicycle	U	U	U	U	U
Car	U	U	U	U	U
Motorcycle	U	U	U	U	U
School bus	U	U	U	U	U
Public bus	U	U	U	U	U
Metro/Train/Tram	U	U	U	U	U
Others: ______________ (please specify)	U	U	U	U	U

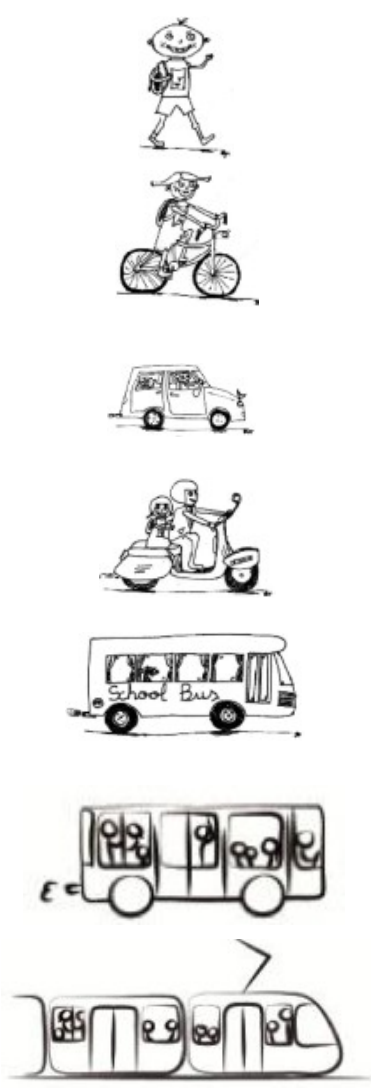

10. What do I think about my usual way of travelling to school?

		Totally agree (1) …… agree (5)				Totally disagree
		1	2	3	4	5
**10.1.**	My usual way of getting to school suits me fine.	U	U	U	U	U
**10.2.**	I feel capable of walking or cycling to the educational centre………………	U	U	U	U	U
**10.3.**	I feel very comfortable when I go to school. accompanied by…………………………………………………………………........................	U	U	U	U	U
**10.4.**	The way I travel to school fits perfectly because I want to go…………………………………………………………………………….	U	U	U	U	U
**10.5.**	I have the necessary skills to travel on foot or by bicycle to educational centre without problems……………………………………………………………	U	U	U	U	U
**10.6.**	I interact very amicably with those around me when I go to the educational centre……………………………………………………………	U	U	U	U	U
**10.7.**	The way I travel to school is whatever I feel like...	U	U	U	U	U
**10.8.**	I am able to travel to the educational centre on foot or by bicycle...	U	U	U	U	U
**10.9.**	I feel that I can communicate openly with those around me accompany……………………………………………………………………………………….	U	U	U	U	U
**10.10.**	I can choose how to travel to school……………………………	U	U	U	U	U
**10.11.**	I feel capable of travelling to school by bicycle or on foot……………………………………………………………………………………….	U	U	U	U	U
**10.12.**	I feel very comfortable with those accompanying me.	U	U	U	U	U

Proyecto PACO $$$$ Put the code here!

0

11. I walk or cycle to school...

		Nothing dero (0)	...Total	mind	truth	ro (4)
		0	1	2	3	4
**11.1.**	Because others tell me I should...	U	U	U	U	U
**11.2.**	Because I feel guilty when I don't do it...	U	U	U	U	U
**11.3.**	Because I value the benefits of walking to school or by bicycle.	U	U	U	U	U
**11.4.**	Because I think walking or cycling to school is fun.	U	U	U	U	U
**11.5.**	Because they agree with my way of being..	U	U	U	U	U
**11.6.**	I don't know why I should walk or cycle to school...	U	U	U	U	U
**11.7.**	Because my friends/family/teacher tell me I have to do it.	U	U	U	U	U
**11.8.**	Because I feel embarrassed if I don't do it	U	U	U	U	U
**11.9.**	Because it is important for me to walk or cycle to school regularly..	U	U	U	U	U
**11.10.**	Because I believe that walking or cycling to the city centre is part of who I am	U	U	U	U	U
**11.11.**	I don't see why I should bother walking or cycling to school...	U	U	U	U	U
**11.12.**	Because I enjoy walking or cycling to school...............	U	U	U	U	U
**11.13.**	This is a control question, mark option 3.....................................	U	U	U	U	U
**11.14.**	Because other people will be unhappy with me if I don't walk or cycle to school...	U	U	U	U	U
**11.15.**	I don't see the point of walking or cycling to school...............	U	U	U	U	U
**11.16.**	Because I see that walking or cycling to school is a fundamental part of who I am..	U	U	U	U	U
**11.17.**	Because I feel like I have failed when I have not walked or cycled to school one day.	U	U	U	U	U
**11.18.**	Because I think it is important to make the effort to walk or cycle to school regularly..	U	U	U	U	U
**11.19.**	Because I find walking or cycling to school an enjoyable activity..	U	U	U	U	U
**11.20.**	Because I feel pressure from my friends/family to walk or cycle to school...	U	U	U	U	U
**11.21.**	Because I believe that walking or cycling to school is in line with my values...	U	U	U	U	U
**11.22.**	Because I get nervous if I don't walk or cycle to school regularly...	U	U	U	U	U
**11.23.**	Because I like walking or cycling to school................	U	U	U	U	U
**11.24.**	I think walking or cycling to the city centre is a waste of time...	U	U	U	U	U

12. How many days a week do you have Physical Education classes? (<u>Select only one option</u>)

a) U 0 days (never)
b) U 1 day
c) U 2 days
d) U 3 days
e) U 4 days
f) U 5 days (all)

13. How many breaks do you have per day?

a) U 0 (none)
b) U 1
c) U 2
d) U 3
e) U 4

14. In general, do you enjoy physical activity?

a) U Not at all
b) U A little
c) U Some
d) U Quite a lot
e) U Very much

15. Do you enjoy Physical Education classes at school?

a) U Not at all
b) U A little
c) U Somewhat
d) U Quite a lot
e) U Very much

Next, we will ask you about your physical activity **<u>at school</u>**. This includes **Physical Education** classes, but also the activity you do during **breaks**, as well as on the **way** to **school** and on the **way home**. Answer by thinking about the physical activity you have done at school during **<u>the last 7 days.</u>**

16. Going to school: How many days did you walk or cycle to school? (<u>Select only one option</u>. If you do not remember exactly, <u>try to select the most appropriate answer</u>).

a) U 0 days (never)
b) U 1 day
c) U 2 days
d) U 3 days
e) U 4-5 days (every day)

17. Activities during Physical Education classes: how often did you run and move around in games or activities organised by the teacher? (If you did not have Physical Education, select "I did not have Physical Education").

a) U I did not have Physical Education
b) U Very little time
c) U Short time
d) U About half the time
e) U A lot of time
f) U Almost all the time

18. Activities during breaks: How often did you play sports, walk, run or play actively? (If you did not have breaks, select "I did not have breaks at school").

a) U I did not have breaks at school
b) U Very little time
c) U A little time
d) U About half the time
e) U A lot of time
f) U Almost all the time

19. Activity during lunch break: During the midday lunch break in the school canteen, how often were you moving around, walking or playing? (If you did not have lunch in the school canteen, select 'I did not eat at school').

a) U I did not eat at school
b) U Very little time
c) U Short time
d) U About half the time
e) U A lot of time
f) U Almost all the time

20. Returning from school: How many days did you walk or cycle home from school? (Select only one option. If you do not remember exactly, <u>try to select the most appropriate answer</u>).

a) U 0 days (no days)
b) U 1 day
c) U 2 days
d) U 3 days
e) U 4-5 days (every day)

These questions are about your level of **physical activity** at different times (**outside school**). This includes both structured **sports activities** and time spent playing **with** friends, dancing or doing **household chores** (tidying your room, cleaning, etc.). Answer based on the physical activity you have done outside school during **the last 7 days.**

21. Activity before going to school: Before going to school (between 6:00 and 8:30-9:00), how many days did you do physical activity for at least 10 minutes? (This includes activities done at home, but NOT others such as walking or cycling to school.) (Select only one option.)

a) ☐ 0 days (no days)
b) ☐ 1 day
c) ☐ 2 days
d) ☐ 3 days
e) ☐ 4-5 days (every day)

22. Activity after returning from school: After returning from school (between 2:00 p.m. and 2:30 p.m. and 6:00 p.m.), how many days did you engage in physical activity for at least 10 minutes? (This includes activities such as playing with friends and family, team sports, or classes in which you engage in physical activity, but NOT walking or cycling to and from school.) (Select only one option.)

a) ☐ 0 days (no days)
b) ☐ 1 day
c) ☐ 2 days
d) ☐ 3 days
e) ☐ 4-5 days (every day)

23. Afternoon activity: In the afternoons (between 6 p.m. and 10 p.m.), how many days did you engage in physical activity for at least 10 minutes? (This includes activities such as playing with friends and family, team sports, or classes in which you engage in physical activity, but NOT walking or cycling to and from school.) (Select only one option.)

a) ☐ 0 days (no days)
b) ☐ 1 day
c) ☐ 2 days
d) ☐ 3 days
e) ☐ 4-5 days (every day)

24. **Saturday activity: How much physical activity did you do last Saturday? (This includes physical exercise, housework, family outings, sports, dancing, or games. If you don't remember exactly, <u>try to choose the most appropriate answer</u>).**

a) U No physical activity (0 minutes)
b) U Very little physical activity (1-30 minutes)
c) U A moderate amount of physical activity (31-59 minutes)
d) U Lots of physical activity (1-2 hours)
e) U A large amount of physical activity (more than two hours)

25. **Sunday activity: How much physical activity did you do last Sunday? (This includes exercise, chores, family outings, sports, dancing, or games. If you don't remember exactly, <u>try to choose the most appropriate answer</u>).**

a) U No physical activity (0 minutes)
b) U Very little physical activity (1-30 minutes)
c) U A moderate amount of physical activity (31-59 minutes)
d) U A lot of physical activity (1-2 hours)
e) U A great deal of physical activity (more than two hours)

These questions are about the time you spend **resting and sitting.** You are probably sitting while **you eat**, do your **homework** or play **musical instruments**, but you may also be sitting when you watch television, play **video games**, use your **computer**, **mobile phone**, **tablet/iPad** or other devices. Answer these questions by thinking about the time you have spent without moving during these activities in **<u>the last 7 days (only on school days).</u>**

26. **Time watching television: How much time did you spend watching television outside of school? (This includes time spent watching films or sports, but <u>NOT</u> playing video games).**

a) U I did not watch any television
b) U I watched television for less than 1 hour per day
c) U I watched television between 1-2 hours a day
d) U I watched television for more than 2 hours and up to 3 hours a day
e) U I watched television for more than 3 hours a day

27. **Time spent playing video games: How much time did you spend playing video games outside of school? (This includes playing Nintendo DS, Wii, Xbox, PlayStation, or other consoles. Do <u>NOT</u> include computer games.)**

a) U I didn't play any video games
b) U I played less than 1 hour per day
c) U I played between 1-2 hours a day
d) U I played more than 2 hours and up to 3 hours a day
e) U I played more than 3 hours a day

28. **Computer time: How much time did you spend using the computer outside of school? (This does NOT include use for homework, but DOES include social media such as Facebook, surfing the internet, chatting, playing video games or online games).**

a) U I did not use the computer for these activities
b) U I used the computer for less than 1 hour per day
c) U I used the computer between 1-2 hours a day
d) I used the computer for more than 2 hours and up to 3 hours a day
e) U I used the computer for more than 3 hours a day

29. **Mobile phone use: How much time did you spend using your mobile phone outside of school? (This includes time spent talking on the phone and sending text messages or WhatsApp messages. If you do not have a mobile phone and never use your parents' or friends' phones, select the option "I never used a mobile phone").**

a) U I never used my mobile phone
b) U I used my mobile phone for less than 1 hour a day
c) U I used my mobile phone between 1-2 hours a day
d) U I used my mobile phone for more than 2 hours and up to 3 hours a day
e) U I used my mobile phone for more than 3 hours a day

30. **Sedentary habits in a typical week (NOT just the last week): Which of the following statements best describes your sedentary habits at home?**

a) U I hardly ever sit down in my free time
b) U I sit for short periods of time in my free time
c) U I sit for a moderate amount of time in my free time
d) U I sit for a long time in my free time
e) U I sit almost all the time in my free time

31. **From Monday to Friday during the weeks you attend school, how many days do you usually have breakfast?**

a) U I never have breakfast on the days I attend school
b) U 1 day
c) U 2 days
d) U 3 days
e) U 4 days
f) U 5 days

32. **What is the reason you usually skip breakfast?**

a) U I never skip breakfast
b) U I don't have enough time
c) U I don't like the food we have at home
d) U I never remember
e) U I'm not hungry in the mornings
f) U I can't eat early in the morning
g) U Other: ______________________

33. **What do you usually have for breakfast on weekdays before going to school? (Answer yes or no for all foods).**

Food	YES	NO	Food	YES	NO
• Milk	U	Y	• Chocolate without milk	U	U
• Natural yoghurt	U	U	• Milk chocolate, cocoa powder (Cola-cao, Nesquik, etc.) or Nutella	U	U
• Cheese	U	U	• Nuts	U	U
• Natural fruit pieces (banana, apple, orange, etc.) or natural juice	U	U	• Olive oil	U	U
• Fruit juice in cartons or fruit in syrup	U	U	• Butter or margarine	U	U
• White bread (loaf or tin, oatmeal, corn, etc.)	U	U	• Dairy desserts (flavoured milkshakes, yoghurts or sweetened or fatty drinking yoghurts, custards, flan, soya-oat drinks)	U	U
• Wholemeal bread (loaf or tin, oat, corn, etc.)	U	U	• Pastries (Maria biscuits, Tosta Rica, Príncipe, Oreo, etc. Muffins, sponge cakes, croissants, Neapolitan pastries, etc.)	U	U
• Cereals without added sugar or oat flakes	U	U	• Serrano ham, cooked ham, chicken/turkey breast cold cuts, etc.	U	U
• Breakfast cereals (Chocapic, Nesquik, Kellogg's, etc.)	U	U	• Eggs (fried, scrambled, boiled, omelette, etc.)	U	U
• Sugar (more than 1 tablespoon)	U	U	• Pâtés, foie gras, other fatty cold cuts (mortadella, salami, chorizo, salchichón, fuet)	U	U
• Honey (up to one tablespoon)	U	U	• Tomato, avocado, carrot, etc.	U	U
• Jam, marmalade, etc.	U	U			

34.1. **A what time do do you usually go to bed on days before class?**

34.2. **At what time do you do you usually wake up on school days?**

24-hour format

Time you go to bed

$$: $$

0U	0U	0U	0U
1U	1U	1U	1U
2U	2U	2U	2U
	3U	3U	3U
	4U	4U	4U
	5U	5U	5U
	6U		6U
	7U		7U
	8U		8U
	9U		9U

Wake-up time

$$: $$

0U	0U	0U	0U
1U	1U	1U	1U
2U	2U	2U	2U
	3U	3U	3U
	4U	4U	4U
	5U	5U	5U
	6U		6U
	7U		7U
	8U		8U
	9U		9U

35. Is the area where you live pleasant for walking or cycling?

	**The area where I live:**	Strongly disagree disagree	Somewhat disagree	Somewhat agree	Very much agree
1	Offers a pleasant environment for walking and cycling	U	U	U	U
	**In the area where I live:**	None	A few	Quite a few	Many
2	The buildings are clean and free of graffiti	U	U	U	U
3	There are quite a few trees on the streets	U	U	U	U
4	There are poorly maintained, empty or ugly buildings	U	U	U	U

36. It is difficult for me to walk or cycle to school because:

		Strongly disagree (1) ……Strongly agree (4)			
		1	2	3	4
a.	There are no pavements or cycle lanes………………………………………………….	U	U	U	U
b.	The road is boring…………………………………………………………….	U	U	U	U
c.	The road is not well lit.	U	U	U	U
d.	There are one or more dangerous intersections.	U	U	U	U
e.	I feel too hot and sweat, or it always rains...	U	U	U	U
f.	Other children do not walk or cycle…………………………..	U	U	U	U
g.	It is not considered cool to walk or cycle………………………..	U	U	U	U
h.	I'm carrying too much stuff………………………………………….	U	U	U	U
i.	It is easier to drive or be driven...	U	U	U	U
j.	Too much advance planning is required…………………………….	U	U	U	U
k.	There are no places to safely leave your bicycle…………………	U	U	U	U
l.	There are stray dogs………………………………………………………………	U	U	U	U
m.	It's too far away.	U	U	U	U
	I would have to walk/cycle through areas that would be unsafe due to crime or other crime-related issues (e.g. vandalism, graffiti, people drinking alcohol in public places)……………………………………….	U	U	U	U
o.	I do not enjoy walking or cycling to school....	U	U	U	U
p.	There are too many hills.	U	U	U	U
q.	There is too much traffic……………………………………………………………….	U	U	U	U
r.	The cycle lanes are occupied by people walking.	U	U	U	U

37.1. Who do you go to school with? **37.2. Who do you return from school with? (You may select multiple options)**

a. By myself............................... ∪
b. With my mother.............................. ∪
c. With my father............................. ∪
d. With my grandmother............................ ∪
e. With my grandfather............................ ∪
f. With other children/teenagers... ∪
g. With other adults....................... ∪

a. By myself .... ∪
b. With my mother .... ∪
c. With my father........................... ∪
With my grandmother............................ ∪
e. With my grandfather............................ ∪
f. With other children/adolescents... ∪
g. With other adults....................... ∪

38. If you are usually accompanied by an adult (father, mother, grandparent, etc.), explain why by answering all the questions with yes or no. If you go alone or with other young people, skip to question 39.

	YES	NO
1. I live far from the school..	∪	∪
2. My parents won't let me go alone..	∪	∪
3. I don't like going alone..	∪	∪
4. There is too much traffic..	∪	∪
5. I'm carrying a lot of weight in my rucksack...	∪	∪
6. It suits my parents because they drop me off on their way to work...	∪	∪
7. It suits my parents because I have younger siblings.	∪	∪
8. Other:________________________________	∪	∪

39. If you could choose:

39.1. How would you like to travel to school? **39.2. How would you like to return from school?**

(Select only one option)

1. On foot........................... ∪
2. Cycling............................. ∪
3. Car................................. ∪
4. Motorbike.................................. ∪
5. School bus................ ∪
6. Public bus................ ∪
7. Metro/train/tram.......... ∪
8. Scooter............................. ∪
9. Other:____________________ ∪

1. On foot............................. .∪
2. Bicycle.............................. ∪
3. Car.................................. ∪
4. Motorbike................................... ∪
5. School bus.................. ∪
6. Public bus.................. ∪
7. Metro/train/tram............ ∪
8. Scooter............................... ∪
9. Others:____________________ ∪

REMEMBER THAT IT IS VERY IMPORTANT TO HAVE ANSWERED ALL THE QUESTIONS AND NOT TO LEAVE ANY BLANK.

PLEASE CHECK THAT EVERYTHING IS COMPLETED!

40. **Do you have any suggestions/comments? Let us know!**

End time (24-hour format) $$: $$			
0U	0U	0U	0U
1U	1U	1U	1U
2U	2U	2U	2U
	3U	3U	3U
	4U	4U	4U
	5U	5U	5U
	6U		6U
	7U		7U
	8U		8U
	9U		9U

CONGRATULATIONS, YOU'RE DONE!

THANK YOU FOR YOUR PARTICIPATION
