# Supplementary material for: Are sleep and breakfast behaviours predictors of active or independent commuting to school in Spanish youth?
Source: BMC Public Health. 2026 Aug 22;26:2415. doi: 10.1186/s12889-026-29160-9 (PMC13499306; doi:10.1186/s12889-026-29160-9)
Supplement: Supplementary file 2 [file 12889_2026_29160_MOESM1_ESM.docx]

**Figure S1.** ROC curve (AUC = 0.850) for the model predicting the mode of commuting to school based on daily breakfast consumption in children.

**Figure S2.** ROC curve (AUC = 0.857) for the model predicting the mode of commuting to school based on sleeping duration in children.

**Figure S3.** ROC curve (AUC = 0.813) for the model predicting the mode of commuting to school based on daily breakfast consumption in children (boys).

**Figure S4.** ROC curve (AUC = 0.832) for the model predicting the mode of commuting to school based on sleep duration in children (boys).

**Figure S5.** ROC curve (AUC = 0.832) for the model predicting the mode of commuting to school based on daily breakfast consumption in children (girls).

**Figure S6.** ROC curve (AUC = 0.832) for the model predicting the mode of commuting to school based on sleep duration in children (girls).

**Figure S7.** ROC curve (AUC = 0.876) for the model predicting the mode of commuting to school based on daily breakfast consumption in adolescents.

**Figure S8.** ROC curve (AUC = 0.875) for the model predicting the mode of commuting to school based on sleep duration in adolescents.

**Figure S9.** ROC curve (AUC = 0.882) for the model predicting the mode of commuting to school based on daily breakfast consumption in adolescents (boys).

**Figure S10.** ROC curve (AUC = 0.882) for the model predicting the mode of commuting to school based on sleep duration in adolescents (boys).

**Figure S11.** ROC curve (AUC = 0.880) for the model predicting the mode of commuting to school based on daily breakfast consumption in adolescents (girls).

**Figure S12.** ROC curve (AUC = 0.881) for the model predicting the mode of commuting to school based on sleep duration in adolescents (girls).

**Figure S13.** ROC curve (AUC = 0.698) for the model predicting the independent mobility to school based on daily breakfast consumption in children.

**Figure S14.** ROC curve (AUC = 0.695) for the model predicting the independent mobility to school based on sleep duration in children.

**Figure S15.** ROC curve (AUC = 0.737) for the model predicting the independent mobility to school based on daily breakfast consumption in children (boys).

**Figure S16.** ROC curve (AUC = 0.734) for the model predicting the independent mobility to school based on sleep duration in children (boys).

**Figure S17.** ROC curve (AUC = 0.739) for the model predicting the independent mobility to school based on daily breakfast consumption in children (girls).

**Figure S18.** ROC curve (AUC = 0.741) for the model predicting the independent mobility to school based on sleep duration in children (girls).

**Figure S19.** ROC curve (AUC = 0.815) for the model predicting the independent mobility to school based on sleep duration in adolescents.

**Figure S20.** ROC curve (AUC = 0.814) for the model predicting the independent mobility to school based on sleep duration in adolescents.

**Figure S21.** ROC curve (AUC = 0.770) for the model predicting the independent mobility to school based on sleep duration in adolescents (boys).

**Figure S22.** ROC curve (AUC = 0.771) for the model predicting the independent mobility to school based on sleep duration in adolescents (boys).

**Figure S23.** ROC curve (AUC = 0.847) for the model predicting the independent mobility to school based on daily breakfast consumption in adolescents (girls).

**Figure S24.** ROC curve (AUC = 0.848) for the model predicting the independent mobility to school based on sleep duration in adolescents (girls).

**Figure S25.** Sleep duration and breakfast consumption with Active Commuting to School in the study by Villa-González et al (2019).

**Figure S26.** Sleep duration and breakfast consumption with Active Commuting to School in the study by Martín-Moraleda et al (2023).
